# Updating UK clinical guidelines to incorporate the placebo effect in peri-operative care: challenges and opportunities

**DOI:** 10.3389/fpain.2026.1638970

**Published:** 2026-02-12

**Authors:** Emma Gregory

**Affiliations:** Faculty of Life Sciences & Medicine, King’s College London, London, United Kingdom

**Keywords:** analgesia, pain management, peri-operative management, placebo effect, placebo effect on pain

## Introduction

Placebos are treatments that are pharmacologically inert (1), but whose administration can produce therapeutic psychosocial effects, known as placebo effects (2). In recent years, the placebo effect has come to be increasingly appreciated for its potential and scientific significance: a clinically relevant phenomena with neurobiological foundations and tangible effects on the brain and body (3). Research into potential applications is underway across a range of indications (4, 5). A key area for consideration of clinical use of placebos is analgesia (6), with placebo-mediated effects on pain and relevant biological underpinnings well-established.

Widespread use of placebos in primary care has been reported via an anonymous UK survey, whilst 15% of Swedish surgeons reported utilising surgeries they believe to have “large placebo components” (7, 8). However, placebos are not formally used outside of clinical trial settings in the UK, with no guidance available on their use. Consequently, placebos remain an underutilised tool despite their potential to improve patient outcomes in a range of clinical settings, including peri-operative care.

The “peri-operative” period refers to the duration between the moment of contemplation of surgery until recovery (9). Analgesia used peri-operatively is frequently in the form of opioids; whilst highly effective analgesics, their use may be associated with immediate risks such as respiratory depression (10). Consequently, strategies that could reduce overall opioid requirements, such as multi-modal analgesia, are a focus of current research (11). Given that medical advances often emerge in unexpected and transformative ways, there is no reason to assume that current approaches represent the limits of what is possible. Here, evidence regarding the potential clinical applications of the placebo effect within peri-operative care are explored, alongside considerations and current barriers to inclusion in UK clinical guidelines.

## Potential applications

### Pre-operative care

The placebo effect incorporates both biological effects and a sizeable psychological component, of which expectancy is the most important factor (12). The value of expectancy has been demonstrated within pre-surgical care; optimism pre-operatively is a significant predictor of pain intensity over up to eight weeks post-coronary artery bypass graft [CABG; (13)]. Furthermore, more optimistic patients also experienced fewer physical symptoms than expected post-CABG. Whilst conclusions cannot be extrapolated to other surgeries, they may be applicable to revascularisation surgeries such as percutaneous coronary intervention, notably used as the first-line intervention for myocardial infarction (14). These data suggest that nurturing positive expectations pre-surgery can promote better recovery.

Positive expectancy has also been associated with an apparent doubling of the analgesic benefit of remifentanil [a potent opioid licensed for use in surgery (15)] in healthy volunteers (16). While intriguing, these results were derived from a small sample size and relied heavily on fMRI measures, which are subject to methodological limitations (17). Thus, although the 20-point difference in pain ratings between positive and negative expectancy groups provides the foundation for further investigation, these findings should be interpreted with caution and require replication in larger patient populations with clinically relevant endpoints.

The expectancy component of the placebo effect could be utilised as an adjunct to pharmacological intervention via additional pre-surgical counselling, fostering a positive expectation of the surgery and recovery process. This could aid in minimising patient anxiety, which further shapes expectation (18). Given that pre-operative anxiety affects around half of surgical patients (19), this represents an area in which post-operative wellbeing and outcomes could be improved. However, such therapy would require careful balance with the need to give a comprehensive and accurate summary of likely treatment outcomes to ensure informed consent can be obtained (20). Consequently, “optimism counselling” would be contraindicated in surgeries with higher morbidity and mortality (21).

### Surgery

Although largely beyond the scope of this review, it is important to acknowledge evidence suggesting that some of the therapeutic benefits of surgery may be partially attributable to placebo effects (8, 22, 23). A systematic review found no statistically significant difference in outcomes between patients undergoing interventional surgery and those receiving sham surgery, which omits the component believed to be therapeutically active for the pain condition in question (22, 24).

Sham surgeries have also been associated with fewer adverse effects compared to interventional procedures (23), raising the possibility that they may offer a safer and potentially efficacious alternative. However, the disparity in adverse event rates raises concerns about whether sham surgeries serve as appropriately matched controls. Thus, while sham surgery studies are valuable in demonstrating placebo components of surgical benefit, their interpretation requires caution. Furthermore, whilst such procedures may appear to offer practical benefits such as reduced hospital stays, psychological burden (25), healthcare costs and wait times (26), and fewer workdays lost to recovery (27), these claims should be regarded with caution. It remains unclear to what extent these outcomes are genuinely attributable to placebo effects rather than other factors, and further evaluation is needed.

### Post-operative care

Early studies of the placebo effect focused on post-operative pain modulation, with evidence dating back to the 1980s (28). Consequently, the efficacy of concealed placebo in reducing post-operative pain is well-established, and the focus of modern research has shifted towards open-label placebo to circumvent the ethical issues experienced with concealed placebo (see Ethical considerations). Post-spinal surgery patients reported that open-label placebo with positive conditioning reduced daily morphine usage by 30% over 17 days, and the worst pain scores of patients in the open-label group were numerically lower vs. those receiving usual treatment (29). Furthermore, the concept of open-label placebo was generally well-received by patients who had undergone hand surgery, suggesting that patients may be open to the use of placebo in this context (30). This is of particular significance given the importance of mindset and positivity in fostering a placebo-mediated improvement in pain (13, 16, 18).

However, the study reported only a 1-point reduction in peak daily pain and a non-significant 0.8-point decrease in average daily pain compared with controls (29). These findings confirm that, while open-label placebo may hold some promise, the evidence base is currently too limited to draw firm conclusions about clinical applications, and further studies are warranted. Despite the small cohort size, further trials are underway, including studies of open-label placebo for reducing post-operative opioid use in post-spinal surgery patients and for acute pain in healthy volunteers (31, 32).

It is imperative that patient pain is suitably controlled by placebo and that the reduction in conventional analgesia does not come at the detriment of patient comfort. As with pre-operative care, decisions would be made on a surgery-specific basis in conjunction with patients' wishes. However, current evidence indicates that adjunctive open-label placebo may in future be a viable option for post-operative analgesia.

### Current limitations to establishing relevant clinical guidelines in the UK

Clinical guidelines are evidence-based (33, 34), hence treatments must be supported by a wealth of data regarding their efficacy and safety before recommendation. Guidelines are informed by the best available science, with well-designed randomised clinical trials (RCTs) considered the gold standard (35–37). However, RCTs are not without limitations, several of which are of particular concern when considering placebo effects. Inclusion in a RCT relies on enrolment triggering participation bias; studies advertised as investigating placebo effects or novel approaches to pain perception may selectively attract individuals who are more open to new ideas and/or the placebo effect (38). Given the importance of mindset in the placebo effect (13, 18), the conclusions drawn from RCTs may not be generalisable. Despite the need for informed consent for participation (39), a carefully articulated justification that highlights uncertainty about how these mechanisms may be harnessed for clinical benefit, and that acknowledges the potential for altruistic contribution to future patients, may increase willingness to enrol among at least some potential participants (40, 41). Furthermore, mitigation of factors that may deter patients, such as concerns regarding exacerbation of disease, could also aid recruitment (42).

Subtle differences in approaches of investigators within RCTs and variation in bedside manner and warmth may also impact patient anxiety and expectancy (38). Whilst this is universally true of RCTs with multiple investigators, this is of particular significance when investigating the placebo effect given the impact of anxiety and expectancy (13, 16, 18). Additionally, many of the studies investigating the efficacy of placebo effects are performed in healthy volunteers (31, 38). Whilst useful proof of concept, there are structural and functional neurobiological changes which occur in the brains of patients with chronic pain and illnesses which may not be reflected in healthy volunteers (43). As such, extrapolating these findings to clinical populations involves a significant inferential leap, and further research in patient cohorts is necessary to establish clinical relevance.

It is also essential to recognise the value of opioids in pain management. Several analytic reviews demonstrate that opioid-based therapy is generally safe and effective for the majority of patients subgroups (44–46). These perspectives highlight that opioids remain an indispensable component of pain management, and that placebo-mediated analgesia should be viewed as complementary to, rather than a replacement for, established pharmacological approaches. Furthermore, risks associated with opioid therapy are not evenly distributed across patient populations: patients with inadequately treated pain compounded by psychiatric comorbidities represent a subgroup at particularly elevated risk of adverse outcomes (46). This heterogeneity reinforces the complexity of opioid prescribing and the limitations of “one size fits all” approaches to analgesia.

Studies investigating placebo-mediated effects often examine the difference between pain scores in the placebo and standard of care arms at a key timepoint, with a statistically significant difference deemed to indicate the superiority of placebo (13, 16, 31, 38). However, statistical significance is not a suitable metric from which to draw conclusions regarding clinical significance, which is ultimately the best of measure of whether efficacy of a treatment translates to improvements in a patient's quality of life and functional status (47). When considering outcomes, it is important to use measures that can determine whether treatments will have a worthwhile impact on patient experiences. Without suitably designed trials, guidelines and policymakers will likely ignore any therapeutic value of the placebo effect.

### Ethical considerations

There are ethical issues associated with concealed placebos (35, 48). Doctors have a moral and legal responsibility to provide details of available treatments prior to treatment initiation, allowing them to make an informed decision (20). Whilst the moral principle of beneficence and acting in the best interests of the patient is also a key consideration in clinical practice, it does not supersede the legality of informed consent (49). It would not be acceptable for doctors to deceptively prescribe a placebo for pain relief in the belief that it is the most efficacious option available, thus patient autonomy remains an understandable barrier to the use of blinded placebos in peri-operative care.

Many of the ethical drawbacks associated with the use of placebos can be circumvented via open-label placebos, for which there is increasing evidence. Unlike concealed placebo administration, open-label placebos maintain transparency with patients and respect for autonomy, yet may still derive meaningful benefit. However, the current evidence base is limited: most existing trials involve small sample sizes and frequently recruit healthy volunteers rather than surgical or chronic pain patients (31, 38). Moreover, the magnitude and durability of open-label placebo effects in real-world clinical contexts remain uncertain. These limitations emphasise the need for further well-designed studies in patient populations where perioperative pain management is most clinically relevant.

## Conclusion

In conclusion, existing studies provide intriguing but preliminary evidence that placebo mechanisms may have potential clinical applications within the peri-operative timeline. However, the current evidentiary base remains scant, heterogeneous and often limited to proof-of-concept studies in healthy volunteers. It would therefore be premature to draw firm conclusions about clinical applicability. Further rigorous research in surgical patient populations, with clinically meaningful endpoints will be essential before integration into UK perioperative guidelines can be considered. Whilst the placebo effect may ultimately represent a useful adjunct in perioperative pain management, current findings provide limited evidence that require much further investigation in clinical populations.
